# The Potential Relationship Between Environmental Endocrine Disruptor Exposure and the Development of Endometriosis and Adenomyosis

**DOI:** 10.3389/fphys.2021.807685

**Published:** 2022-01-28

**Authors:** Victoria R. Stephens, Jelonia T. Rumph, Sharareh Ameli, Kaylon L. Bruner-Tran, Kevin G. Osteen

**Affiliations:** ^1^Department of Obstetrics and Gynecology, Women’s Reproductive Health Research Center, Vanderbilt University School of Medicine, Nashville, TN, United States; ^2^Department of Pathology, Microbiology and Immunology, Vanderbilt University School of Medicine, Nashville, TN, United States; ^3^Department of Microbiology and Immunology, Meharry Medical College, Nashville, TN, United States; ^4^VA Tennessee Valley Healthcare System, Nashville, TN, United States

**Keywords:** endometriosis, adenomyosis, environmental toxicants, endocrine disrupting chemical (EDC), inflammation

## Abstract

Women with endometriosis, the growth of endometrial glands and stroma outside the uterus, commonly also exhibit adenomyosis, the growth of endometrial tissues within the uterine muscle. Each disease is associated with functional alterations in the eutopic endometrium frequently leading to pain, reduced fertility, and an increased risk of adverse pregnancy outcomes. Although the precise etiology of either disease is poorly understood, evidence suggests that the presence of endometriosis may be a contributing factor to the subsequent development of adenomyosis as a consequence of an altered, systemic inflammatory response. Herein, we will discuss the potential role of exposure to environmental toxicants with endocrine disrupting capabilities in the pathogenesis of both endometriosis and adenomyosis. Numerous epidemiology and experimental studies support a role for environmental endocrine disrupting chemicals (EDCs) in the development of endometriosis; however, only a few studies have examined the potential relationship between toxicant exposures and the risk of adenomyosis. Nevertheless, since women with endometriosis are also frequently found to have adenomyosis, discussion of EDC exposure and development of each of these diseases is relevant. We will discuss the potential mechanisms by which EDCs may act to promote the co-development of endometriosis and adenomyosis. Understanding the disease-promoting mechanisms of environmental toxicants related to endometriosis and adenomyosis is paramount to designing more effective treatment(s) and preventative strategies.

## Introduction

**FIGURE 1:**
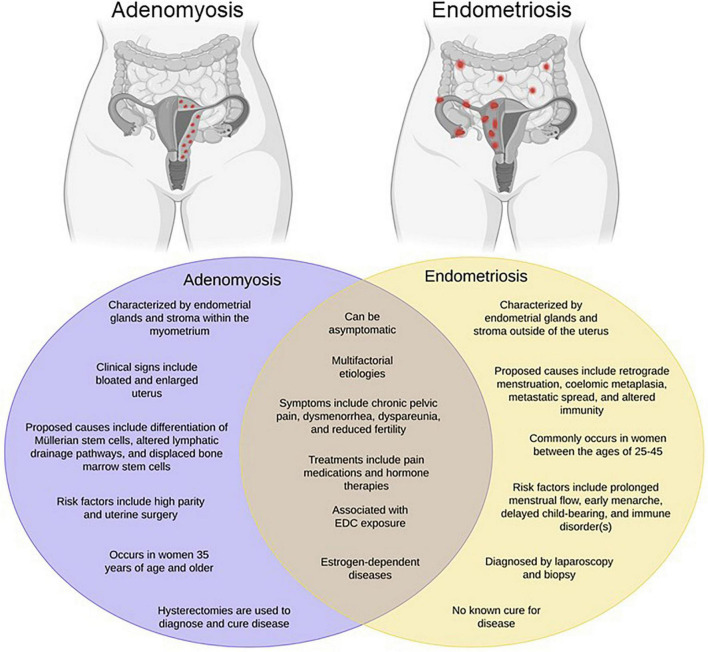
Endometriosis and adenomyosis exhibit overlapping phenotypes. Adenomyosis has been referred to as “endometriosis interna” due to its resemblance to endometriosis both histologically and phenotypically. However, as shown in the Venn diagram, while the diseases have many common features, they also exhibit a number of differences. Created with BioRender.com.

Commonly occurring as comorbidities, endometriosis and adenomyosis affect millions of reproductive-aged women worldwide, yet remain two of the most poorly understood gynecologic diseases. Endometriosis is best characterized as the growth of endometrial glands and stroma at extra-uterine sites, whereas adenomyosis, often times referred to as “endometriosis interna,” is defined as the presence of endometrial glands and stroma embedded within the uterine muscle Figure 1. Despite the proposition of numerous theories, the precise etiology of either disease remains unknown. One of the oldest and most accepted hypotheses for endometriosis is Sampson’s theory of retrograde menstruation (Sampson, 1927). Although ectopic attachment of refluxed menstrual debris remains a plausible mechanical explanation for endometriosis development, it cannot account for all incidences of disease occurrence given that the majority of cycling women exhibit this phenomenon, yet only a subset develop disease (Halme et al., 1984). Additional theories regarding the development of endometriosis include coelomic metaplasia, activation of stem cell rests, and inherent epigenetic abnormalities (Gruenwald, 1942; Baranova et al., 1999; Figueira et al., 2011; Sourial et al., 2014; Bulun et al., 2019).

At present, several theories regarding the pathogenesis of adenomyosis have also been proposed. One theory hypothesizes that adenomyosis emerges from the invagination of the basalis region of the endometrial mucosa into the myometrium as a result of disturbances to the endometrial junctional zone, a steroid-dependent region situated between the endometrium and myometrium (Brosens et al., 1995; Bergeron et al., 2006; Sofic et al., 2016; Vannuccini et al., 2017; Bulun et al., 2021). The endometrial-myometrial interface lacks a mucosa-muscle tissue layer; as a result, the endometrium sits on top of the myometrium, thereby providing an opportunity to invade the myometrium at sites of weakened smooth muscle fibers (Uduwela et al., 2000). An alternative theory suggests that adenomyosis evolves through *de novo* metaplasia of tissues from embryologic pluripotent Müllerian ducts (Garcia-Solares et al., 2018). Müllerian tissues, composed of glands and stroma, are vital for the maturation of the female urogenital system including the development of fallopian tubes, cervix, uterus, and superior vagina (Wilson and Bordoni, 2021). Observations and studies of adenomyotic lesions revealed both biological and proliferative properties that are distinct to Müllerian tissues, including the cytokeratin filaments and vimentin characteristic of Müllerian epithelial tissue and mesenchymal tissue, respectively (Moll et al., 1983; Matsumoto et al., 1999). Another theory hypothesizes that adenomyosis develops as a result of the inevitable mechanical strain of the uterus due to its continuous remodeling activity throughout the reproductive period of a female’s life (Leyendecker et al., 2009, 2015). Like many other tissues, mechanisms involved in mechanical strain, tissue injury, and tissue repair stimulate local production of estrogen. Hyperestrogenism results in peristalsis of the myometrium which inflicts supraphysiological strain on cells at the fundo-cornual raphe (Leyendecker et al., 1998, 2004; Garavaglia et al., 2015). These events initiate the tissue injury and repair system further increasing and prolonging the production of estrogen. Importantly, deviation from the normal hormonal cycle and hyperestrogenism is suggested to be a cause of uterine dysfunction in women with adenomyosis and endometriosis (Leyendecker et al., 2009). Despite the numerous theories that have been proffered to explain the development of adenomyosis, our current understanding of disease pathogenesis remains limited. Not surprisingly, the lack of mechanistic understanding of the disease limits the development of treatment options for women with adenomyosis. Although hysterectomy is considered curative for adenomyosis since it is localized to the uterine muscle, this is not an option for women desiring to preserve pregnancy. In these patients, like most women with endometriosis, fertility-sparing treatments are typically designed for management of disease symptomology and are not curative.

In 2017, it was reported that 42.3% of women (*n* = 300) diagnosed with endometriosis also had adenomyosis (Antero et al., 2017). An additional study designed to address the co-occurrence of disease observed a significant increase in the presence of focal adenomyosis located in the outer myometrium in women with endometriosis (50.2%) compared to women without the latter disease (5.4%) (Chapron et al., 2017). Using magnetic resonance imaging, Kishi et al. (2012) identified four subtypes of adenomyosis, defined as due to direct endometrial invasion (Subtype I), endometriotic invasion from the outer uterus (Subtype II), *de novo* metaplasia (Subtype III) and heterogeneous, advanced disease of mixed phenotype (Subtype IV). Interestingly, this study reported that 96% of women with Subtype II adenomyosis also had endometriosis, while only 15% of women with subtype I had coexisting endometriosis (Kishi et al., 2012). In a follow-up study, this same group presented data suggesting that subtype II adenomyosis arises as a consequence of pelvic endometriosis (Kishi et al., 2017). In this regard, our laboratory has identified deep, adenomyotic lesions in our mouse model of developmental toxicant exposure also presenting with an endometriosis-like uterine phenotype (Nayyar et al., 2007; Bruner-Tran et al., 2016). Examining whether early life exposure to endocrine-disrupting chemicals affects the frequent occurrence of these diseases as co-morbidities in women may provide a unique insight for future therapies.

Collectively, the above studies highlight the possibility of a common or overlapping disease pathway for endometriosis and adenomyosis. Hence, it is important to identify underlying causes that promote the development of either disease to improve patient reproductive health outcomes. Many women with endometriosis and/or adenomyosis experience chronic episodes of debilitating pain, dyspareunia, dysmenorrhea, and/or infertility. Regardless of whether endometriosis and adenomyosis are two separate entities or different manifestations of the same disease, both human and animal studies have implicated a role for inflammatory processes in their development (Jiang et al., 2016; Orazov et al., 2016; Lacheta, 2019; Vannuccini and Petraglia, 2019; Maruyama et al., 2020; Bulun et al., 2021). For example, ectopic establishment of endometrial tissues is associated with the induction of an inflammatory peritoneal environment which negatively impacts uterine steroid responsiveness (Stilley et al., 2009; Bruner-Tran et al., 2018; Sharpe-Timms et al., 2020). Together, the local and systemic hyperinflammatory environment, in conjunction with the altered hormonal milieu associated with endometriosis, is suggested to promote the subsequent development of adenomyosis.

Environmental toxicants are ubiquitously present in the environment and also have the potential to promote disease as a consequence of immune or endocrine-disrupting actions (Diamanti-Kandarakis et al., 2009; Sutton et al., 2012; Wang et al., 2016; Street et al., 2018). Not surprisingly, environmental toxicants that act as endocrine-disrupting chemicals (EDCs) have been implicated in the pathogenesis of both endometriosis and adenomyosis (Bruner-Tran et al., 2016, 2017). Both diseases are estrogen-dependent, and numerous EDCs have been identified as estrogen mimics that are capable of inducing adverse effects mediated through both ER-dependent and ER-independent signaling pathways. Therefore, it is important to determine if exposure to common EDCs is among the underlying triggers for endometriosis and adenomyosis, and if so, how can we use this knowledge to prevent these diseases and/or improve patient outcomes. Herein, we will consider the available evidence and will also preset possible mechanisms by which EDCs may promote the development of disease.

## Environmental Endocrine Disrupting Chemicals

The endocrine system is an assembly of hormone-secreting tissues that promote downstream interactions between hormones and their highly specific receptors to regulate essential life processes including growth and development, tissue function, metabolism, and reproduction (Hiller-Sturmhofel and Bartke, 1998). EDCs are classically defined as exogenous chemicals that interfere with the normal physiological functions of the endocrine system potentially causing adverse health effects and promotion of disease (Rumph et al., 2020). Unfortunately, due to industrialization, exposure to EDCs is inevitable and occurs across the lifespan of most mammals. Human exposures can occur via encounters with consumer products packaged in material that leach EDCs as well as through consuming contaminated foods (e.g., meat and dairy products). Production of manmade chemicals, many with endocrine-disrupting actions, began in the early- to mid-1900s when manufacturers saw their benefits as plasticizers, pesticides/insecticides, and medications. By the end of the 20th century, studies began to unveil the harmful effects associated with exposure to individual EDCs (Schug et al., 2016). Although not all EDCs have been assessed for their toxicity and effects on human health, several EDCs have been evaluated resulting in the reduction and eventual ban of certain compounds: polychlorinated biphenyls (PCBs), dichloro-diphenyl-trichloroethane (DDT), and diethylstilbestrol (DES; reviewed by Schug et al., 2016).

Endocrine disrupting chemicals can be divided into two groups: persistent organic pollutants (POPs) and non-persistent EDCs (npEDCs) Table 1. npEDCs have a low lipid solubility resulting in a relatively short half-life in humans and animals whereas POPs are highly lipophilic chemicals that are not readily biodegradable (Cho et al., 2020). Development of a variety of reproductive diseases has been found to be associated with both npEDCs (Kandaraki et al., 2011; Ye et al., 2018; Bariani et al., 2020; Peinado et al., 2021) and POPs (Heilier et al., 2004; Bruner-Tran and Osteen, 2010; Trabert et al., 2015; Al Jishi and Sergi, 2017). Due to their lipophilic nature, a number of EDCs, including dioxins and polychlorinated biphenyls (PCBs), are ubiquitously present in the environment despite regulatory steps to curtail production and manufacture. Furthermore, EDCs such as TCDD and benzo(a)pyrene (BaP), are unintentionally released as byproducts of both natural and industrial processes; thus, human exposure is difficult to avoid. Due to their lipophilic nature, numerous EDCs are capable of penetrating the lipid bilayer of plasma membranes fostering their bioaccumulation in adipose tissue and enhancing their ability to bio-magnify within the food chain. Following the initial exposure, adipose tissue slowly releases EDCs into the blood stream, thereby contributing to the potential for long-term adverse health effects.

**TABLE 1:** Health effects of selected endocrine disrupting chemicals.

**Type of endocrine disrupting chemicals**	**Endocrine disrupting chemicals**	**Sources of exposure**	**Industrial benefits**	**Potential gynecologic health risks**	**References**
Persistent EDCs	Dioxins	Combustion, waste incineration, volcanic eruptions, forest fires	N/A	Endometriosis, adenomyosis, reproductive cancers	Fouzy et al. (2007), Bruner-Tran and Osteen (2010), Bruner-Tran et al. (2016), Kishi et al. (2017)
	Polychlorinated Biphenyls (PCBs)	Electrical transformers, microscope immersion oils, pesticides, carbonless copy paper	Electrical insulating compounds	Endometriosis, adenomyosis, uterine fibroids	Rier et al. (2001), Heilier et al. (2004), Trabert et al. (2015)
Non-persistent EDCs	Bisphenol A (BPA)/ Bisphenol S (BPS)	Children’s toys, water bottles, canned food liners, dental sealants, receipt coatings	Plasticizer and epoxy resins	Endometriosis, uterine fibroids, polycystic ovarian syndrome, adenomyosis	Newbold et al. (2007), Cobellis et al. (2009), Kandaraki et al. (2011), Bariani et al. (2020)
	Phthalates	Cosmetics, medical equipment, medications, paints, adhesives, personal care products	Plasticizers, solvents, and stabilizers	Endometriosis, uterine fibroids, adenomyosis	Duty et al. (2005), Huang et al. (2010), Bariani et al. (2020)
	Parabens	Cosmetics, pharmaceutical products	Preservatives	Endometriosis, uterine fibroids	Bariani et al. (2020), Peinado et al. (2021)
	Triclosans (TCSs)	Hand sanitizers, mouth wash, toothpaste	Antimicrobial properties	Polycystic ovarian syndrome	Ye et al. (2018)

Many EDCs mediate their effects by binding with high affinity to the aryl hydrocarbon receptor (AhR), an orphan nuclear receptor that is expressed throughout the female and male reproductive tracts and by cells of the immune system (Brockmoller et al., 1994; Hernandez-Ochoa et al., 2009; Jiang et al., 2010; Stockinger et al., 2014). When inactive, AhR is localized to the cytoplasm (Noakes, 2015). Upon activation by ligand binding, AhR is released from the translocation inhibitory complex (formed by chaperone proteins HSP90 and XAP2) which reveals AhR’s nuclear localization signal (NLS). Once the NLS is exposed, AhR is translocated to the nucleus where it forms a heterodimeric complex with the aryl hydrocarbon receptor nuclear transporter (ARNT; Humphrey-Johnson et al., 2015). Within the nucleus, this complex acts a transcription factor that interacts with the xenobiotic response element (XRE) within the promoter region of numerous genes (Humphrey-Johnson et al., 2015; Noakes, 2015). Importantly, AhR/ARNT transcriptional activities are dependent upon the conformational structure of the bound receptor which is suggested to be directly influenced by ligand binding (Fujii-Kuriyama and Kawajiri, 2010; Denison and Faber, 2017; Larigot et al., 2018). The AhR engages in the modulation of various cellular processes, including differentiation, apoptosis, and proliferation, when bound appropriately by endogenous ligands (De Coster and van Larebeke, 2012). Conversely, inappropriate activation of the AhR by exogenous ligands can have wide-ranging detrimental effects that may promote diseases such as endometriosis and adenomyosis (Bulun et al., 2000; Bock and Kohle, 2006).

In addition to AhR, EDCs are also able to mediate their actions through nuclear receptor subfamily 1, group I, member 2 (NR1I2), also known as the steroid and xenobiotic receptor (SXR). Like AhR, SXR is an orphan nuclear receptor that regulates the metabolism and cellular response to xenobiotics compounds, such as EDCs and pharmaceuticals (Zhou et al., 2009). Although quite similar, AhR typically binds dioxin-like compounds and SXR typically binds non-dioxin like compounds (Egusquiza et al., 2020). EDCs are characterized as dioxin-like compounds or non-dioxin-like compounds based on their similarities in structure and mechanism of action to 2,3,7,8-tetrachlorodibenzo-p-dioxin (TCDD), the most potent EDC/POP currently known to man (Van den Berg et al., 2006).

### 2,3,7,8-Tetrachlorodibenzo-p-Dioxin

2,3,7,8-tetrachlorodibenzo-p-dioxin (TCDD) is a persistent and ubiquitously present environmental toxicant that is released as a byproduct of both industrial (e.g., burning of fossil fuels and paper bleaching) and natural processes (e.g., volcanic eruptions and forest fires). Like most EDCs, TCDD is lipophilic in nature and highly resistant to degradation. TCDD is capable of modulating signaling processes mediated by both estrogen and progesterone, steroid hormones required for the maintenance of normal uterine physiology. TCDD exposure has been experimentally linked to the development of reproductive disorders in mammals, including endometriosis. This association was first reported in a landmark study conducted by Rier et al. (1993) which revealed a positive correlation between TCDD exposure and the incidence of endometriosis in a colony of rhesus monkeys (Rier et al., 1993). This study sparked a surge in subsequent studies that sought to examine the potential link between TCDD exposure and the development of endometriosis (Bois and Eskenazi, 1994; Eskenazi et al., 2002; Heilier et al., 2005; Matta et al., 2021). Nevertheless, epidemiologic data is mixed with a number of studies failing to identify a clear association between TCDD exposure and endometriosis (reviewed in Rumph et al., 2020).

Numerous experimental studies conducted in rodents have more definitively linked EDC exposure to reproductive disease development. Our laboratory utilizes a developmental toxicant exposure model created by exposing pregnant C57/BL6 mice (F0, or founding generation) to a single 10 μg/kg dose of TCDD by oral gavage on embryonic day 15.5 providing *in utero* and lactational exposures of TCDD to offspring (F1 generation) (Bruner-Tran et al., 2017). Additionally, germ cells present in the F1 feti have the potential to become the F2 generation and are also directly exposed during pregnancy. Subsequent generations (F3 and beyond) are indirectly exposed by way of a familial history. Although mice do not menstruate and do not spontaneously develop endometriosis, studies have revealed an endometriosis-like uterine phenotype in mice with a history of TCDD exposure that is markedly similar to that of women with endometriosis. More specifically, our laboratory identified a reduced expression of proteins required for the establishment and maintenance of pregnancy in the offspring of the F0 generation (F1 mice) including progesterone receptor and progesterone-regulated transforming growth factor- beta 2 (TGF-β2; Nayyar et al., 2007). In a separate study, we found that approximately 50% of F1 females were infertile. Of those who achieved pregnancy, we noted an increased rate of spontaneous preterm birth compared to unexposed, pregnant control female mice (Bruner-Tran and Osteen, 2011; Ding et al., 2011). Equally important, we found that the endometriosis-like phenotype persisted in up to three generations following a single TCDD exposure to the F0 dam.

With regard to adenomyosis, we are aware of only two studies that have examined TCDD exposure and the presence of disease. In our laboratory, we retrospectively assessed uteri from multiple generations of TCDD exposed mice (F1–F3 animals described above) for evidence of adenomyosis. While none of the uteri from control mice showed evidence of adenomyosis, we identified advanced disease denoted by deep, adenomyotic lesions in mice with a direct (F1–F2) and indirect (F3) *in utero* TCDD exposure. We found that 70% (*n* = 10) of F1 females, 63% (*n* = 11) of F2 females, and 56% (*n* = 9) of F3 females exhibited histological and pathological evidence of deep adenomyosis (Bruner-Tran et al., 2016). Studies from another laboratory described uterine changes consistent with adenomyosis following a short-term dioxin exposure to Baladi goats. Specifically, adult female goats were exposed to nonane (vehicle) or a mixture containing 17 PCDDs and PCDF congeners (0.23 μg/kg body weight) in nonane three times over 9 days and euthanized 16 days after the final dose. Animals exposed to the dioxin mixture exhibited degenerative and necrotic changes associated with inflammatory reaction in liver and kidney as well as uterine adenomyosis (Fouzy et al., 2007).

Taken together, these data provide evidence that EDC exposure can promote the development of both endometriosis and adenomyosis in multiple species. However, while numerous studies support a role of TCDD in the development of endometriosis in women (reviewed by Rumph et al., 2020), to our knowledge, a potential role of this compound and the development of adenomyosis in women has not been investigated.

### Phthalates

Phthalates are anti-androgenic and estrogenic EDCs that can be found in plastics, medical devices, and children’s toys. Because a large quantity of phthalates are added to feminine care products and cosmetics, women are typically at risk for higher levels of exposure compared to men (Duty et al., 2005). In relation to the presence of disease, phthalates are present at significantly higher concentrations in plasma of women with endometriosis (Upson et al., 2013). More specifically, Korean and Indian women with advanced disease were found to have significantly higher levels of mono-ethylhexyl phthalate level (MEHP) and di-(2-ethylhexyl) phthalate (DEHP) in their plasma compared; to disease-free women (Reddy et al., 2006a,b; Hartmann, 2020). In two additional studies, the National Health and Nutrition Examination Survey (NHANES) and the Endometriosis, Natural History, Diagnosis, and Outcomes study, results also revealed a significant association between urinary phthalates and endometriosis (Weuve et al., 2010; Buck Louis et al., 2013). Furthermore, studying the association between phthalate exposure and the presence of disease in Taiwanese women revealed a significant increase (*p* < 0.05) in urinary mono-n-butyl phthalate (MBP) in patients with endometriosis (Huang et al., 2010). Similar results were seen in an additional study from this group that identified a modest increase in urinary MEHP in Taiwanese patients with either endometriosis or adenomyosis (Huang et al., 2014).

### Bisphenols

Bisphenols are estrogen-mimicking EDCs that are capable of maintaining low levels of progesterone receptors which can lead to disruptions in uterine cyclicity, a potential mechanism for the development of endometriosis (Aldad et al., 2011). To date, bisphenol A (BPA), previously used in the manufacturing of food cans and dental sealants, is one of the most well-studied and widespread EDCs, and it is abundantly present in sera of women with endometriosis compared to women without disease (Brotons et al., 1995; Olea et al., 1996; Cobellis et al., 2009; Rashidi et al., 2017). Upson et al. (2014) conducted a population-based case-control study to determine if BPA exposure was linked to an increased risk of endometriosis. After measuring total urinary BPA concentrations in 143 cases (women with surgically diagnosed endometriosis) and 287 controls (women without a known endometriosis diagnosis), this study revealed a statistically significant, positive correlation between urinary BPA concentrations and peritoneal endometriosis, but not ovarian disease (Upson et al., 2014). In contrast to the previous study, patients with ovarian endometriomas were found to have significantly higher urinary BPA concentrations than controls (Rashidi et al., 2017); conversely, other studies found no association between urinary BPA concentrations and endometriosis (Itoh et al., 2007; Buck Louis et al., 2013). Inconsistencies among human studies likely reflect differences in populations, experimental design variations, and the rigorousness of the control groups (Buck Louis et al., 2013).

Since endometriosis is an estrogen-dependent disease, Jones et al. (2018) sought to determine the effects of BPA and bisphenol AF (BPAF; a more estrogenic bisphenol than BPA) on the progression of disease with and without the influence of endogenous and exogenous estrogen. To create their mouse model of endometriosis, minced uterine tissue from donor mice was injected into the peritoneal cavity of hormonally intact and ovariectomized mice and allowed to freely attach, similar to human disease. Six weeks after the injection and consuming the study diet [containing vehicle, ethinylestradiol (EE), BPA, or BPAF], lesions, ovaries, and blood were collected and examined for endometriosis-associated characteristics (number of lesions, lesion weight, and lesion volume) (Jones et al., 2018). Although total lesion number was not different between groups, total lesion weight and volume was increased in EE-treated ovariectomized mice. Similarly, total lesion number was unchanged regardless of EDC exposure in intact mice. EE treatment of ovariectomized mice did not alter lesion weight; however, a significant increase in lesion volume was observed in EE-treated mice that also received BPA or BPAF. Furthermore, investigators isolated RNA from endometriotic lesions in the peritoneal cavity of mice in each experimental group to investigate the effects of EDC exposure on the gene expression of known estrogen/estrogen receptor mediated targets [e.g., *progesterone receptor (PGR) and lactoferrin*] (Burns et al., 2012; Jones et al., 2018). As expected, ovariectomized mice treated with EE had a greater expression of *PGR* and *lactoferrin* than vehicle-treated mice, and EDC-treated mice had a significantly lower expression of *PGR*. As for the hormonally intact mice, *PGR* and *lactoferrin* significantly increased following EE treatment (Jones et al., 2018). These data are significant because it is established that estrogen maintains the expression of the PGR. It is also known that while estrogen-mimicking EDCs are capable of binding to the estrogen receptor, these EDCs are unable to produce normal downstream processes resulting in the lack of expression of downstream targets such as the PGR. This work further demonstrates the estrogen-dependent nature of endometriosis, and that progression/maintenance phase of disease is also hormone-dependent.

In a study conducted by Signorile et al. (2010) they demonstrated that prenatal BPA-exposure in mice induces an endometriosis-like ectopic disease identified by the presence of endometrial glands and stroma in adipose tissue adjacent to the reproductive tract (Signorile et al., 2010). This study suggests that parental EDC exposure predisposes female offspring to reproductive diseases such as endometriosis, and potentially, adenomyosis. Although the association of BPA exposure and adenomyosis has not been well studied, Newbold et al. (2007) identified a link between neonatal BPA-exposure and the development of severe pathologies of the female reproductive tract, including adenomyosis. Female pups were treated from post-natal day 1 (PND1) to PND5 with varying concentrations of BPA (10, 100, or 1000 μg/kg/day). After 18 months, their uteri showed dose-dependent, histological evidence of adenomyosis (6% controls, 9% BPA-10, 20% BPA-100, and 19% BPA-1000) (Newbold et al., 2007). Nevertheless, data linking BPA exposure to the development of adenomyosis in women is limited compared to evidence in the literature suggesting that BPA, and related compounds, can promote endometriosis. Therefore, knowing that endometriosis is suggested to predate adenomyosis makes the aforementioned studies relevant to understanding the relationship between BPA exposure and disease development.

### Medications as Endocrine Disruptors

#### Diethylstilbestrol

One of the most well-known pharmaceutical EDC exposures was as a consequence of diethylstilbestrol (DES) consumption by pregnant women in an attempt to mitigate the risk of miscarriage, preterm birth, and other pregnancy-related complications (Rumph et al., 2020). DES is a synthetic, highly potent estrogen that was initially prescribed to women with high-risk pregnancies. Soon after, it was recommended to all pregnant women from the 1940s through the 1970s. In 1971, DES was banned in the United States because, in addition to being completely ineffective in preventing pregnancy loss, it was found to increase the risk of serious disease in both the mothers and their children (Noller and Fish, 1974; Herbst et al., 1999; Harris and Waring, 2012; Reed and Fenton, 2013). Relevant to the current discussion, additional studies revealed an increased incidence of endometriosis in women whose mothers were prescribed DES compared to the daughters of women that were not given DES during pregnancy (Brosens et al., 2014; Benagiano et al., 2015; Upson et al., 2015). Although epidemiology studies have yet to identify *in utero* DES exposure as a risk factor for the development of adenomyosis, animal studies suggest that there is a positive association between the two. Huseby and Thurlow (1982) fed pregnant mice a 0.2 μg/g DES-containing diet beginning on embryonic day 7 until PND1. Upon maturity, female offspring exhibited significantly reduced fertility and fecundity. Furthermore, they developed adenomyotic lesions that resembled the human disease (Huseby and Thurlow, 1982).

#### Hormonal Contraceptives

Contraceptives are often used by women to prevent the occurrence of pregnancy and/or mitigate the symptoms of reproductive diseases. Although the increased exposure to exogenous estrogen could enhance the development of adenomyosis, the use of low dose estradiol formulations can also be effective in decreasing endogenous production of this steroid, potentially reducing disease risk (Upson and Missmer, 2020). Despite the fact that several studies have attempted to examine the impact of hormonal-based contraceptives on the development of adenomyosis, a consensus has yet to be reached. For example, two studies examined the incidence of adenomyosis in women who underwent hysterectomy and found no association between the presence of disease and history of hormonal contraceptive use (oral contraceptives and intrauterine device) (Parazzini et al., 1997, Parazzini et al., 2009). On the contrary, Templeman et al. (2008) conducted a population-based cohort study in which a significant association between contraceptive use and adenomyosis emerged. Of the women in this study who previously and/or currently use oral contraceptives, 84% had endometriosis and 80% had adenomyosis. Moreover, women who formerly used oral contraceptives were 54% more likely to have a pathology-confirmed adenomyosis diagnosis (POR = 1.54, 95% CI = 1.28-x-1.85) (Templeman et al., 2008). Results from this study suggests a history of oral contraceptive use is positively associated with the presence of adenomyosis; however, it unclear whether these women used contraceptive methods for birth control or to treat symptoms of disease (e.g., heavy menstrual bleeding and pelvic pain). Therefore, it is possible that adenomyosis-related symptoms lead to contraceptive use as opposed to contraceptive use being a risk factor for disease development.

#### Tamoxifen

Tamoxifen is a non-steroidal estrogen receptor modulator with strong anti-estrogenic effects in breast tissue and thus is used to treat postmenopausal breast cancer in women. Tamoxifen has also been found to have tumorigenic effects in the female reproductive tract, hence its association with endometrial carcinoma, sarcoma, polyps, and hyperplasia (Cohen et al., 1995; Nasu et al., 2008). Being that tamoxifen can influence endometrial tissue, tamoxifen treatment has been identified as a risk factor for the development of adenomyosis (Taran et al., 2013; Struble et al., 2016; Upson and Missmer, 2020). Not surprisingly, postmenopausal, tamoxifen-treated breast cancer patients are more likely to develop adenomyosis than similar patients who did not receive tamoxifen (Ugwumadu et al., 1993; Cohen et al., 1995, 1997; Ascher et al., 1996). The first and largest case series study following postmenopausal breast cancer patients revealed that the incidence of adenomyosis in tamoxifen-treated patients was approximately four times the rate reported for premenopausal and non-treated postmenopausal women (Cohen et al., 1995). This research group subsequently conducted an analytical study using postmenopausal women with a history of tamoxifen treatment for breast cancer (*n* = 28) and comparable women without the history of tamoxifen treatment (*n* = 11). No statistical difference was found between any tested parameters between the groups except for the statistically significant rate of adenomyosis diagnosis between groups (*p* = 0.019). Specifically, 53% of patients in the tamoxifen-treated group were diagnosed with adenomyosis whereas only 18.2% of non-tamoxifen treated patients received a histologically confirmed diagnosis (Cohen et al., 1997). In addition to human data, animal studies have also revealed an association between tamoxifen exposure and the subsequent development of adenomyosis (Parrott et al., 2001; Green et al., 2005; Mehasseb et al., 2009; Jin et al., 2020).

## Potential Mechanisms

### Genetic Variations

Recent studies have begun to examine the potential role of either genetic polymorphisms and/or gene-environment interactions in the development of estrogen-dependent diseases. For example, in a study described above, Huang et al. (2010) revealed an increased risk for adenomyosis in women carrying the glutathione S-transferase M1 polymorphism (*GSTM1*) who were also exposed to high levels of phthalates (Huang et al., 2010). *GSTM1* is present as active (GSTM1*A and GSTM1*B) or null (GSTM1*0) alleles, and its protein product is a xeno-compound-metabolizing enzyme that aids in the detoxification of both exogeneous and endogenous compounds (Brockmoller et al., 1994; Baranova et al., 1997). Despite not knowing the true origin of disease for endometriosis and adenomyosis, gene-environment interactions are suggested to play a role in the pathogenesis of environmentally induced diseases. Supporting this concept, individuals lacking *GSTM1* have an increased risk of both lung and bladder cancers, two diseases that have strong environmental associations (Seidegard et al., 1990; Vineis and Simonato, 1991; Viscoli et al., 1993; Brockmoller et al., 1994). Relevant to our interest, Baranova et al. (1997) revealed that none of the patients with endometriosis had the genotype corresponding to high enzymatic activity (*GSTM1A/B*), while 86% of them were *GSTM1*-deficient (*GSTM10/0* genotype). In a subsequent study, this group also identified significant differences in the arylamine N-acetyltransferase 2 (*NAT2*) gene polymorphisms between disease-free women and patients with endometriosis. Like *GSTM1*, *NAT2* encodes an enzyme that is key in the detoxification system, NAT2. The enzymatic activity of NAT2 can be divided into two categories: rapid acetylators and slow acetylators (SA; Cascorbi et al., 1995; Chen et al., 2007). Patients with endometriosis (60%) were found to have significantly higher percentages of SA genotypes in comparison to disease free women (38.9%) suggesting that genetic polymorphisms may predispose individuals to the development of endometriosis and/or adenomyosis (Baranova et al., 1999). The impact of these changes may be exacerbated when present in association with environmental exposures.

Similar to endometriosis, the genomic characteristics of adenomyosis remain unknown. However, Inoue et al. (2019) discovered recurring somatic mutations of oncogenic *KRAS* in adenomyotic lesions harvested from patients. Relevant to our interest, identical *KRAS* mutations were also found to exist in both adenomyotic and endometriotic lesions from several patients with co-occurring disease (Inoue et al., 2019). Using targeted deep sequencing, some of the same genetic mutations found in the lesions (e.g., *KRAS* and *PIK3CA*) were identified in normal, unaffected endometrium adjacent to lesions. These findings raise the possibility that acquired mutations in normal endometrium are driving forces behind the development of adenomyosis. These mutations are also suggested to be responsible for the enhanced invasive capacity of the endometrium which allow endometrial glands and stroma to embed itself within the myometrium. This theory is in agreeance with a previously proposed hypothesis stating that the development of endometriosis is consequent of the retrograde flow of normal endometrial cells with increased proliferative and invasive capacities due to acquired somatic mutations in cancer-associated genes (Suda et al., 2018). Although a few EDCs have been shown to induce genetic mutations, the majority are unable to alter DNA sequences. Conversely, as will be discussed below, epigenetic modifications have frequently been associated with EDC exposure and should be considered with regard to disease risk (Anway et al., 2005; Jirtle and Skinner, 2007; Szyf, 2007; Skinner et al., 2010).

### Epigenetic Alterations

Historically, DNA was thought to be solely responsible for transferring phenotypic information from parent to offspring. Mutations to the DNA is one mechanism by which certain diseases can be transmitted to the next generation. However, we now understand that epigenetic alterations can also induce heritable changes in gene function that are independent of modifications to DNA sequences (Hamilton, 2011). Acquisition of these marks is essential during development, in which epigenetic changes to different cells allows for differentiation along a particular path despite the presence of identical DNA. Indeed, epigenetic modifications during development allows for the unique phenotypic and functional characteristics of specific tissues. Additionally, epigenetic markings accumulate over time and contribute to both aging and the increased susceptibility to age-related diseases. Importantly, epigenetic marks within somatic cells do not affect the phenotype of the next generation. However, epigenetic marks within the germ line have the potential to impact the phenotype of the offspring (Gabory et al., 2011; Bruner-Tran et al., 2012, 2017). Unfortunately, a number of environmental toxicants have been demonstrated to be capable of inducing epigenetic changes within the germ line, leading to multi- and transgenerational effects (Skinner et al., 2010; Bruner-Tran et al., 2012).

There are a variety of mechanisms by which epigenetic modifications can occur. Perhaps the best-studied process for epigenetic modification is DNA methylation at the fifth carbon of the cytosine base, 5-methylcytosine (5mC; Alavian-Ghavanini and Ruegg, 2018). 5mC is predominantly found at cytosine-guanine dinucleotides (CpG)- rich regions (also known as CpG islands) which are commonly found at genomic regulatory regions. Converse to transcriptionally active unmethylated CpGs, methylation of CpG islands generally correlate with a closed chromatin structure which makes DNA inaccessible thereby resulting in transcriptional silencing. DNA methyltransferases (DNMTs), specifically DNMT1, copy the methylation signature from the mother strand to the daughter strand of DNA, hence its maintenance throughout replication and heritability (Moore et al., 2013).

Histone modifications by post-translational modifications (PTMs) are also well-studied in the field of epigenetics. Histones are proteins that bind DNA, provide structural support to chromosomes, and mediate chromatin regulation (Peterson and Laniel, 2004). Chromatin can be found in two states: euchromatin (low binding affinity between the histone and DNA) and heterochromatin (high binding affinity between the histone and DNA) (Tamaru, 2010). The state of chromatin is key in regulating gene expression and it is driven by two types of PTMs: (1) acetylation of lysine at the histone tail resulting in the activation of transcription and (2) methylation of lysine at the histone tail resulting in activation or repression of transcription depending on its position on the histone tail (Bannister and Kouzarides, 2011).

Despite a lack of knowledge regarding the exact mechanistic relationship between EDCs and epigenetic signatures, proposed mechanisms to date can be divided into two categories: gene-specific action and global action (Alavian-Ghavanini and Ruegg, 2018). The bulk of EDC-influenced epigenetic modifications are considered to be gene-specific, and they are suspected to be a consequence of EDC interference of nuclear receptor (NR) function. NRs have been shown to regulate gene-specific chromatin states by engaging histone modifiers and recruiting DNMTs and thymine DNA glycosylases (TDG) to specified genomic loci (Kouzmenko et al., 2010; Martens et al., 2011; Hassan et al., 2017). Although, to our knowledge, direct evidence of an EDC-epigenetic connection has yet to be demonstrated in humans, numerous murine studies indicate that early life EDC exposure is capable of inducing epigenetic modifications. For example, Jefferson et al. (2018) exposed neonatal mice to 1 mg/kg/day DES or vehicle only from PND1-PND5. Their results revealed that pups receiving DES exhibited altered uterine development and impaired reproductive function that was associated with changes to the uterine epigenetic landscape (Jefferson et al., 2018). In our own laboratory, we have found that *in utero* exposure of mice to TCDD was associated with hypermethylation of the PGR *in uteri* of female mice, sperm of male mice, and placentae arising from exposed males (Bruner-Tran et al., 2012; Ding et al., 2018).

Global action characterizes the effects of EDCs on epigenetic regulators, and the most commonly studied are DNMTs. While numerous studies describe the outcome of EDC exposure on the expression of DNMTs, the mechanistic characteristics have yet to be deciphered but are expected to be due to downstream regulation of mRNA and/or miRNA expression by defective receptors (Liu et al., 2013; Derghal et al., 2016). In addition to DNMTs, studies show that EDC exposure deregulates DNA demethylases and histone-modifying enzymes (Jefferson et al., 2013; Karabulut et al., 2016; Kurian et al., 2016; Liu et al., 2016).

As it relates to disease development, Liu and Guo (2012) reported aberrant expression of DNMTs in eutopic endometrium of patients with adenomyosis. Compared to endometrium from women without disease, women with adenomyosis had a significantly higher ectopic expression of DNMT1 (*p* = 3.2 × 10^–6^) and DNMT3B (*p* = 0.002) whereas DNMT3A (*p* = 4.1 × 10^–6^) was significantly reduced in both eutopic and ectopic endometrium. Furthermore, they assessed the relationship between menses, dysmenorrhea, and DNMT expression in patients with adenomyosis. Studies revealed a positive correlation between increased expression of DNMT1 (*p* = 0.015) and menorrhagia, and a positive correlation between increased DNMT3B (*p* = 0.043) and the severity of dysmenorrhea (Liu and Guo, 2012). While studies continue to provide evidence supporting the involvement of epigenetic mechanisms in adenomyosis, additional research is needed to conclusively pinpoint epigenetic aberrations as a mechanism of disease development.

### Steroid Responsiveness

Based on current scientific knowledge, EDCs are able to evoke pathological conditions by interfering with the physiology of hormones controlled by the endocrine system. All hormones execute their functions by interacting with their specific receptors. Not surprisingly, EDCs can act as hormone receptor agonists and/or antagonists resulting in inappropriate receptor activation or inhibition of receptor responses. EDCs are also able to alter hormone receptor expression as seen by the decrease of progesterone receptor expression in the uteri of mice developmentally exposed to TCDD (Nayyar et al., 2007). Downstream signal transductions are consequent of hormone-receptor interactions, and EDCs have been found to perturb both nuclear steroid receptors and cell surface membrane receptors (La Merrill et al., 2020). Perturbation of signal transductions downstream of nuclear receptor activation caused by EDC exposure often include EDC-cofactor interactions. Cofactors (e.g., activators and repressors) are key molecules in the determination of downstream responses produced by receptor activation. The steroid receptor coactivator (SRC) family has received much interest as it relates to the effects of EDC exposure. Relevant to our diseases of interest, binding of xenoestrogens DES and BPA to estrogen receptor (ER)-alpha and ER-beta recruit SRC1 (Routledge et al., 2000). The SRC1-ER complex is suggested to be essential in the early formation of endometriosis as its synergism has been shown to inhibit caspase 8 activation preventing the activation of tumor necrosis factor alpha (TNFα)- induced apoptosis complex II in endometriotic lesions (Han et al., 2015; Chantalat et al., 2020). Not only are EDCs capable of altering hormone synthesis and metabolism as mentioned in a previous section, these compounds are also able to interfere with hormone transport which, as expected, alters the activity of hormone-responsive cells (La Merrill et al., 2020). Taken together, the described modes of action identify how EDCs mediate adverse effects within the endocrine system contributing to diseased phenotypes and pathological conditions.

Endometriosis and adenomyosis are widely known to be estrogen-dependent diseases. Not surprisingly, several studies have identified an association between a hyperestrogenic uterine environment and the presence of disease. Estrogen is biosynthesized through a reaction in which aromatase cytochrome P450 catalyzes the conversion of androgens to estrogens (Simpson and Davis, 2001). In the eutopic endometrium of women with adenomyosis, aromatase cytochrome P450 RNA expression is increased with cytoplasmic localization of the protein in glandular cells as well as in the stroma (Kitawaki et al., 1999). Assessing the utility of aromatase cytochrome P450 mRNA (CYP19) quantity as a diagnostic tool for endometriosis and adenomyosis, Hatok et al. (2011) observed a statistically significant increase (*p* = 0.0002) in CYP19 expression in eutopic endometrial biopsy specimens from women with adenomyosis (56%), endometriosis (73.3%), or both diseases (65.5%) compared to disease-free women (21.7%) (Hatok et al., 2011). Gonadotropin-releasing hormone (GnRH) and danazol are two therapeutic agents that have been shown to induce lesion regression and improve disease-related symptomology by creating a hypoestrogenic state in patients with endometriosis (Dmowski, 1982; Gargiulo and Hornstein, 1997; Selak et al., 2000; Kupker et al., 2002; Ashfaq and Can, 2021). To test the efficacy of these endocrine therapies in the normalization of estrogen metabolism, patients received a GnRH agonist treatment for a minimum of 2 months or a danazol treatment for a minimum of 1 month. Studies revealed that both treatments substantially reduced the aromatase cytochrome P450 mRNA and protein expression in the eutopic endometrium thereby reducing the local production of estrogen (Ishihara et al., 2003). Despite the fact that current research studies have linked atypical steroidogenesis and steroid responsiveness to reproductive disease, further studies are needed to elucidate the mechanistic contribution of steroids to the development of adenomyosis.

### Developmental Origins of Health and Disease

Endometriosis and adenomyosis were historically considered to be idiopathic diseases. More recently, experimental evidence suggests that these diseases may be initiated in the fetal environment (Upson et al., 2015). The concept of “Fetal Origins of Adult Disease” was originally proposed by Dr. David Barker in the 1990s following the identification of increased incidence of metabolic syndrome in adult children of women who experienced severe malnutrition during the Dutch Potato Famine of 1944–1945 (Roseboom et al., 2001; Barker, 2004). The concept of fetal plasticity has evolved as researchers and studies consider other factors, including maternal stress and toxicant exposures, as additional influential contributors to fetal reprogramming (Heindel, 2006; Codagnone et al., 2019; Hsu and Tain, 2019). This expanded concept is now more commonly known as “Developmental Origins of Health and Disease (DOHaD).” This theory postulates that adult health is significantly impacted (both positively and negatively) by the fetal environment. For example, there is an increased risk of significant health-related consequences when exposure to environmental influences such as EDCs occur during critical developmental periods, specifically *in utero*.

**FIGURE 2:**
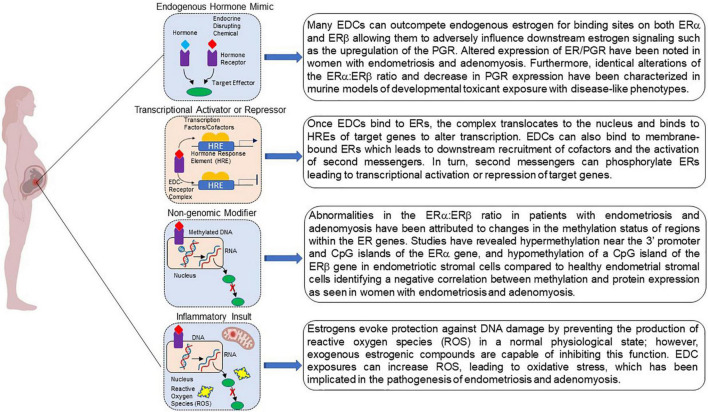
Potential mechanisms by which developmental exposure to environmental endocrine disruptors may induce disease or dysfunction. EDCs may act as steroid agonists or antagonists by binding receptors and interfering with downstream responses. EDCs may also act via non-genomic mechanisms via binding to G-protein coupled receptors. Finally, interference with steroid action can promote inflammation via a variety of mechanisms, including the failure to curtail production of reactive oxygen species. Created with BioRender.com.

Although the link between developmental EDC exposure and subsequent disease occurrence is a topic of ongoing investigation, several mechanisms regarding fetal reprogramming induced by EDC exposure have been proposed Figure 2 (Xin et al., 2015; La Merrill et al., 2020). Because EDCs mimic endogenous hormones and/or block endogenous hormone binding, they are able to mediate their effects through ligand activated mechanisms. EDC-receptor complexes are able to mediate genomic actions by binding to hormone response elements to activate or repress downstream gene transcription. These same complexes are also capable of modifying non-genomic activities by altering signal transduction or introducing post translational modifications yielding epigenome reprogramming. Additionally, EDCs can act by altering the cellular environment as a consequence of inappropriate metabolism and mitochondrial dysfunction evoking subsequent inappropriate downstream signaling cascades that can negatively affect protein function. Together, these mechanisms can increase disease susceptibility.

## Discussion

To date, mechanistic data regarding the early development of adenomyosis are limited; however, evidence within the literature suggests a potential relationship between environmental endocrine disruptor exposure in the development of this disease. Data supporting a role of EDC exposure and the development of endometriosis is more robust, but questions remain due to conflicting reports. As discussed in this review, EDCs are of particular interest in elucidating the etiology of endometriosis and adenomyosis due to their ability to alter steroidogenesis, epigenetic signatures, and immunologic function (Crain et al., 2008; Sifakis et al., 2017; Cho et al., 2020). However, using epidemiological data, proving causality between EDC exposure and human disease has been difficult. Despite a large number of studies and thousands of participants, epidemiological data has failed to consistently link EDC exposure to the development and progression of endometriosis. One reason for the variability may be due to selection biases. Most studies have been conducted using women undergoing hysterectomy for benign conditions (e.g., uterine fibroids) as opposed to identifying women free of gynecologic disease. This methodology presents an issue with linking mechanistic actions of endocrine disrupting chemicals to the risk of disease development since endocrine dysfunction, especially within estrogenic pathways (e.g., menopausal status, menstrual cycle length, early menarche, and contraception use), are typically associated with reasons for hysterectomy (Upson and Missmer, 2020). Another explanation for the inconclusiveness of human studies is that these studies largely examine the link between the adult body burden of EDCs and the incidence of disease without regard to the timing of the initial exposure.

In more recent years, attention has been drawn to the fact that mammals are more susceptible to the effects of EDCs during early life development which illustrates the need to address early life EDC exposure and adult reproductive dysfunction using correlative studies (Crain et al., 2008; Sutton et al., 2010; Meeker, 2012). These studies have suggested a plausible link between EDC exposure and the development of endometriosis, especially in models of early life toxicant exposure. Many women with endometriosis also have adenomyosis; therefore, examination of the endometriosis-focused studies is likely relevant to understanding the contribution of EDC exposures to the etiology and pathogenesis of adenomyosis (Kunz et al., 2005; Leyendecker et al., 2015; Zannoni et al., 2020). While the current research gaps limit our understanding of EDC-induced reproductive dysfunction, developing tools and experimental models to assess the aftermath of developmental toxicant exposure is paramount to formulating treatments as well as methods to block the effects of EDCs and reduce the incidence of reproductive disease development.

## Author Contributions

VS, JR, and SA: researching literature. VS, JR, SA, and KB-T: writing the manuscript. KB-T and KO: final editing and funding. All authors contributed to the article and approved the submitted version.

## Conflict of Interest

The authors declare that the research was conducted in the absence of any commercial or financial relationships that could be construed as a potential conflict of interest.

## Publisher’s Note

All claims expressed in this article are solely those of the authors and do not necessarily represent those of their affiliated organizations, or those of the publisher, the editors and the reviewers. Any product that may be evaluated in this article, or claim that may be made by its manufacturer, is not guaranteed or endorsed by the publisher.
