# Corrigendum: The potential relationship between environmental endocrine disruptor exposure and the development of endometriosis and adenomyosis

**DOI:** 10.3389/fphys.2023.1260961

**Published:** 2023-08-14

**Authors:** Victoria R. Stephens, Jelonia T. Rumph, Sharareh Ameli, Kaylon L. Bruner-Tran, Kevin G. Osteen

**Affiliations:** ^1^ Department of Obstetrics and Gynecology, Women’s Reproductive Health Research Center, Vanderbilt University School of Medicine, Nashville, TN, United States; ^2^ Department of Pathology, Microbiology and Immunology, Vanderbilt University School of Medicine, Nashville, TN, United States; ^3^ Department of Microbiology and Immunology, Meharry Medical College, Nashville, TN, United States; ^4^ VA Tennessee Valley Healthcare System, Nashville, TN, United States

**Keywords:** endometriosis, adenomyosis, environmental toxicants, endocrine disrupting chemical (EDC), inflammation

In the published article, there was an error in the **Funding** statement. The authors omitted the disclaimer, which is required by the U.S. EPA, from their Funding statement. The EPA grant number was also incomplete and was listed as “R839501.”

“This work was supported by the National Institute of General Medical Sciences of the National Institutes of Health under Award Number T32GM007628 (JR), Training Program in Environmental Toxicology under award number TOX T32 ES007028 (VS), Bill and Melinda Gates Foundation (KB-T and KO), and U.S. Environmental Protection Agency #R839501 (KB-T and KO), R01HD096147 (KB-T and KO).”

The correct **Funding** statement appears below.

“The potential relationship between environmental endocrine disruptor exposure and the development of endometriosis and adenomyosis” was developed under Assistance Agreement No. RD83950101 awarded by the U.S. Environmental Protection Agency to KO. It has not been formally reviewed by EPA. The views expressed in this document are solely those of VS, JR, SA, KB-T, and KO and do not necessarily reflect those of the Agency. EPA does not endorse any products or commercial services mentioned in this publication.”

The authors apologize for this errors and state that this does not change the scientific conclusions of the article in any way. The original article has been updated.

